# Reply: Detrimental effects of diabetes mellitus in alcoholic liver disease

**DOI:** 10.1097/HC9.0000000000000855

**Published:** 2026-05-08

**Authors:** Ben Ward, Richard Parker

**Affiliations:** 1 Leeds Liver Unit, St James’s University Hospital, Leeds, UK; 2 Leeds Institute for Medical Research, University of Leeds, Leeds, UK

We would like to extend our gratitude to Drs Akbar and Ahmad for their detailed letter in response to our published manuscript.^1^ We will address their queries in turn.

As we specify in our manuscript, the cohort was built before the consensus statement regarding SLD nomenclature,^2^ so there is an absence of baseline data to completely define metabolic and alcohol-associated liver disease (MetALD). However, as this is an emerging field of research, we considered it valuable and informative to examine the subgroup in the WALDO database where alcohol intake was equivalent to that seen in MetALD. It would be more helpful still if the additional metabolic parameters were available, but unfortunately, this was not possible. We agree that the actual rate of MetALD may have been underestimated if only patients with diabetes mellitus (DM) were considered to have MetALD.

Baseline was defined as the point of liver biopsy. For those cases where a biopsy was performed and a diagnosis of alcohol-associated hepatitis (AH) was given, we are not able to confirm when during their illness the biopsy was performed. We acknowledge that there are likely to be cases where the biopsy was performed during the acute phase of the disease. Although we accept that timing the biopsy during the acute phase of the disease would influence the degree of inflammation, this was not the purpose of the study, and we emphasize how having a cohort of patients with biopsy-proven alcohol-associated liver disease is an advantage to the analysis of the study. Of the patients with AH, only 16 (8.6%) had DM at baseline; too few for meaningful survival analysis. In terms of abstinence from alcohol, our paper includes (table 2) an analysis of the association between abstinence and liver-related death or transplantation, where abstinence was independently associated with better outcomes (HR 0.73, 95 % CI 0.57–0.94, *p*<0.001). While abstinence/non-abstinence did not appear to influence the risk of developing DM in follow-up, we cannot rule out an effect of differing drinking patterns where there may be nuances we could not capture with this binary approach. Similarly, we acknowledge in our manuscript that information regarding the management of DM would be useful, but unfortunately, our data cannot address this.

Data were included from patient records taken from the 1970s and 1980s. While we acknowledge that clinical practice at this time, particularly in the management of DM, was different from current practice, we emphasize that by including data from this far back, we have benefited from having a large cohort as well as a longer timescale in which to record liver-associated clinical events and the development of DM. Both of which help to add further power to the study. The high rates of mortality and transplantation combine to give a relatively short follow-up period, despite the long period of recruitment. Hepatocellular carcinoma (HCC) rates can be captured in short follow-up periods: Ganne-Carrié et al^3^ describe a cumulative incidence of 5.2% in a cohort of patients with cirrhosis and a median follow-up of 29 months. Our cohort includes patients without cirrhosis, and the rates of HCC consequently are lower: we reported a cumulative incidence of 1.8% at 5 years.^4^ A recent large cohort of patients with alcohol use disorder, followed for 10 years, had an overall mortality rate from HCC of 1.2%.^5^ In this latter cohort, the risk of developing liver disease was much higher in the presence of DM, consistent with the point that Drs Akbar and Ahmad raise regarding the natural history of ALD in relation to surveillance for HCC.
